# Case Report: A giant fecaloma revealed by severe aspiration pneumonia and urinary retention

**DOI:** 10.12688/f1000research.54855.1

**Published:** 2021-07-29

**Authors:** Elhem Mchirgui, Hanen Elloumi, Imen Ganzoui, Wissem Triki

**Affiliations:** 1 Emergency Department, Habib Bougatfa hospital, Bizerte, TUNISIA, 7000, Tunisia; 2 Gastroenterology Department, Habib Bougatfa Hospital, BIZERTE, TUNISIA, 7000, Tunisia; 3 Radiology Department, Habib Bougatfa Hospital, BIZERTE, TUNISIA, 7000, Tunisia; 4 Digestive Surgery Department, Habib Bougatfa Hospital, BIZERTE, Tunisia, 7000, Tunisia

**Keywords:** Fecaloma, Elderly, Constipation, CT scan, Complication, Mortality.

## Abstract

Fecaloma is an accumulation of hardened impacted stool typically occurring in the sigmoid colon and rectum. It mainly affects elderly and bedridden patients suffering from chronic constipation and can be revealed by different signs. We report a case of 74-year-old female, with anorexia, Alzheimer’s disease, and chronic constipation, who was admitted to the emergency department with complaints of dyspnea and anuria. Clinical examination showed fever, Glasgow Coma Scale score of 13/15, tachycardia with a blood pressure of 100/50 mmHg, polypnea with hypoxia, foci of crepitant rales in pulmonary auscultation and a tender hypogastric mass with mild diffuse abdominal tenderness. Digital rectal examination revealed hard fecal material. Computed tomography (CT) images demonstrated bilateral pulmonary parenchymal condensation and a huge heterogeneous fecaloma in the sigmoid colon and rectum compressing the bladder. Based on these findings, the diagnosis of giant fecaloma causing aspiration pneumonia and urinary retention was retained. Manual disimpaction and bowel enemas were done but they were unsuccessful and surgical treatment was refused. Ultimately the patient died due to septic shock. Early diagnosis should be made to relieve symptoms and prevent complications.

## Introduction

Fecaloma, a rare cause of fecal impaction, is an accumulation of hardened impacted stool typically occurring in the sigmoid colon and rectum and affecting, especially, elderly patients. ^1– 5^ Symptoms of fecaloma are non-specific, dominated by constipation, abdominal pain, abdominal distention, nausea and vomiting. ^3– 5^ The diagnosis is made through abdominal computed tomography scan (CT scan), X-rays or abdominal ultrasound. ^1, 2^ Fecaloma is often managed conservatively by manual disimpaction, laxatives and enemas. However, endoscopic or surgical interventions may be mandatory especially when it is refractory to conservative treatment or complicated. ^4^ Fecaloma is also associated with increased morbidity and mortality. ^3, 4^ We report a case of a giant fecaloma revealed by severe aspiration pneumonia and urinary retention.

## Case report

A 74-year-old north-African female presented, in March 2021, to the emergency department of our hospital with dyspnea for one week and anuria for one day. The patient had a three-year history of Alzheimer’s disease, anorexia and chronic constipation. For one year, she had been bedridden after a fall. On initial evaluation, she was cachectic, had a Glasgow Coma Scale score of 13/15, a temperature of 38.5°C, a heart rate of 110 beats per minute, blood pressure of 100/50 mmHg and a respiratory rate of 22 breaths per minute with a saturation of 83%. On physical examination, the patient had foci of crepitant rales at both pulmonary bases and mild diffuse abdominal tenderness with a tender hypogastric mass on palpation. Digital rectal examination revealed hard stool impaction.

**Figure 1.**
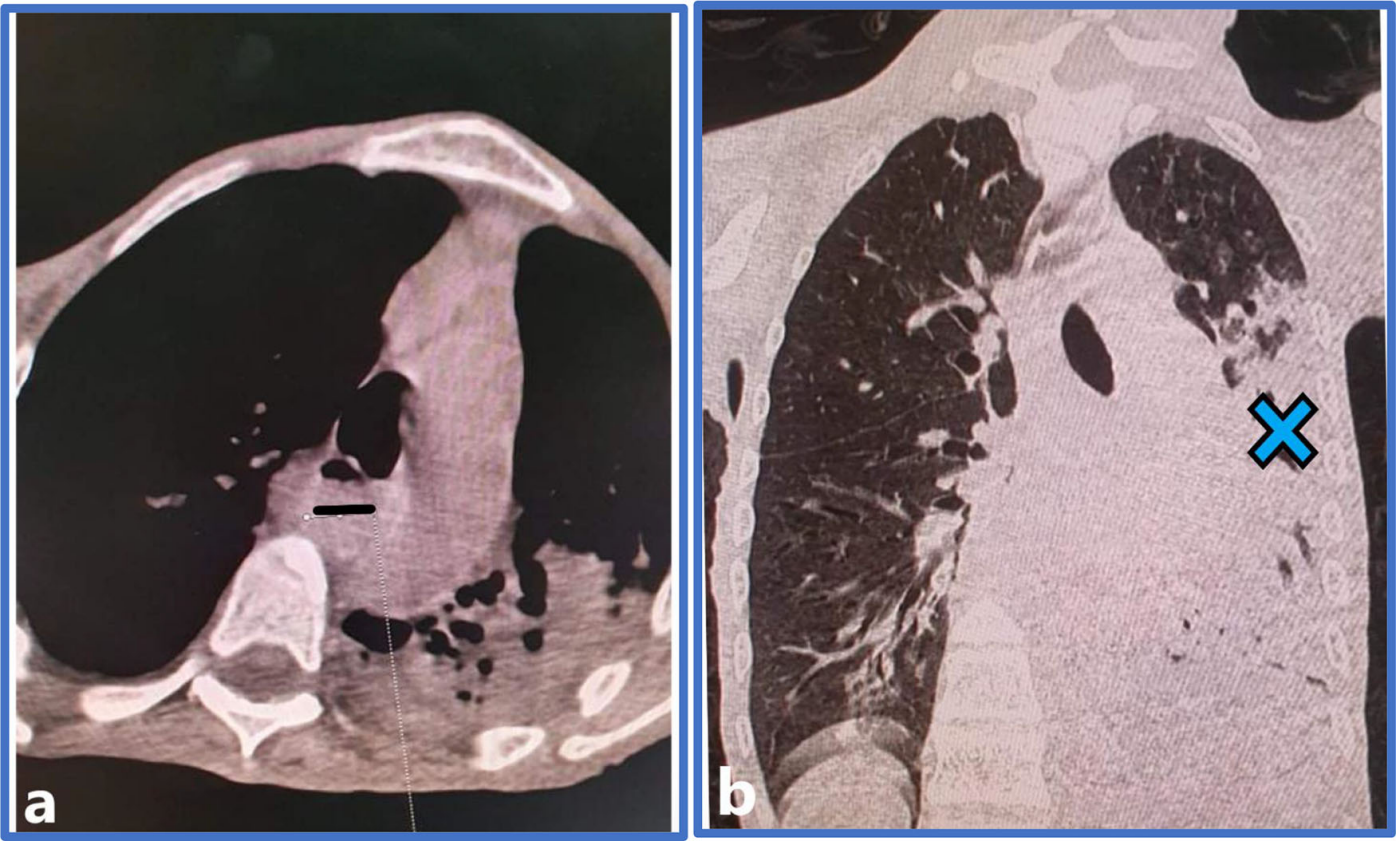
Chest computed tomography with an axial (a) and coronal (b) cuts, revealing esophageal stasis (a, Black line) and pneumonia (b, Blue cross).

**Figure 2.**
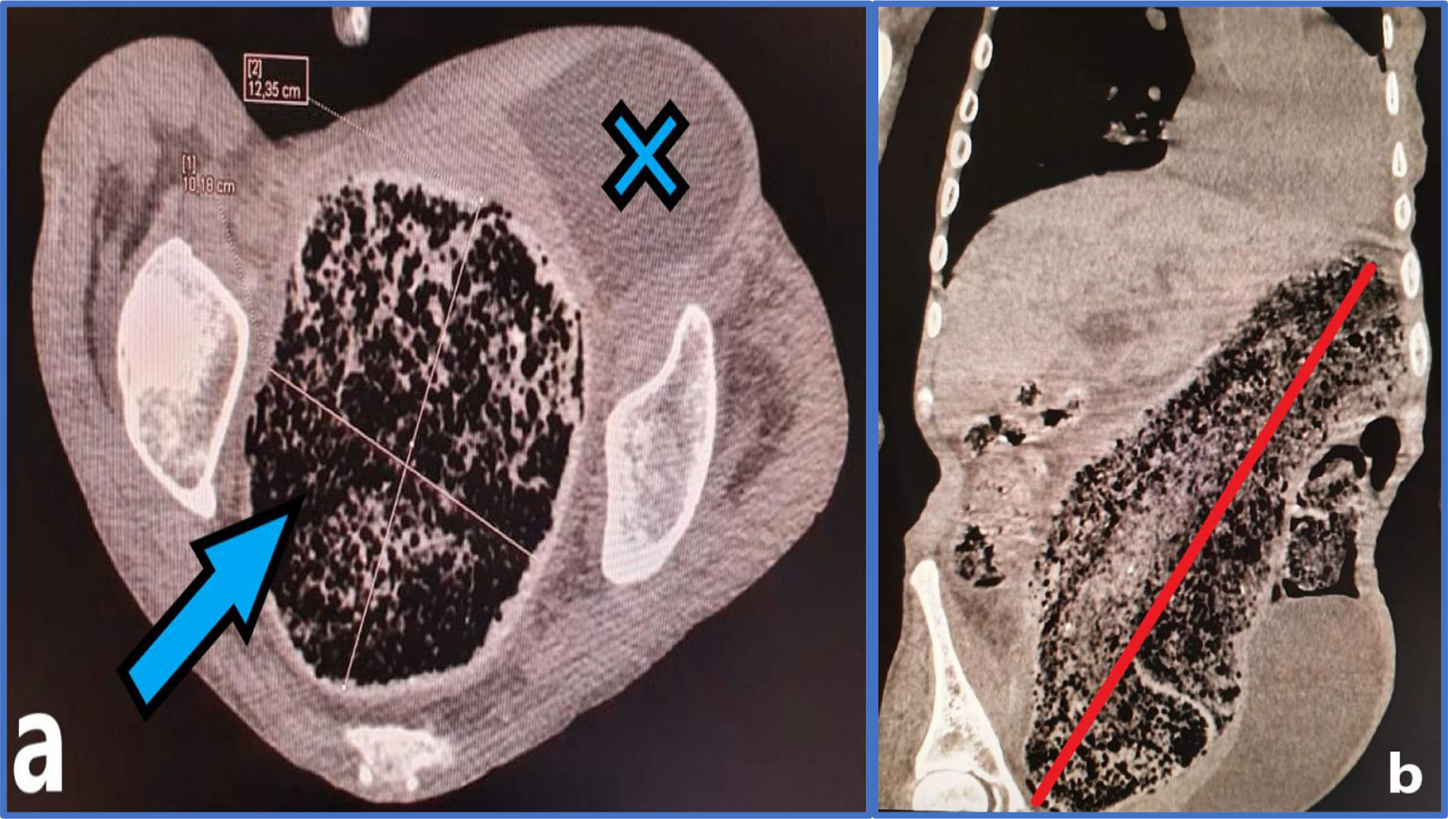
Abdominal computed tomography with an axial (a) and coronal (b) cuts revealing a distension of the rectum and sigmoid colon by a giant fecaloma of 10 × 12 cm (a, Blue arrow) going up to the left hypochondrium (b, Red line). **The bladder is pushed forward and to the left (a, Blue cross).**

Through blood testing, the patient had white blood cells at 5800/mL; hemoglobin at 9.2 g/dL; C-reactive protein at 312 mg/l; urea at 1mg/l; creatinine at 129 μmol/l; natremia at 133 mmol/l; potassium at 4 mmol/l and chloremia at 96 mmol/l. Her blood gas showed respiratory alkalosis with severe hypoxemia at 60 mmHg. Thoraco-abdominal CT scan revealed left pulmonary parenchymal condensation and liquid esophageal stasis ( Figure 1a) suggesting aspiration pneumonia ( Figure 1b) and a huge heterogeneous fecaloma measuring 10 × 12 cm in the sigmoid colon and rectum ( Figures 2a and 2b) associated with stercoral stasis in the remaining colon and compressing the bladder ( Figure 2a). The patient was tested for coronavirus disease 2019 (COVID-19) using a polymerase chain reaction test, due to the pandemic context and the clinical presentation of dyspnea, hypoxemia and pneumonia on the CT scan. The test was negative.

The diagnosis of giant fecaloma causing urinary retention and aspiration pneumonia was retained. A Foley catheter was introduced into the urinary bladder bringing back 500 mL of concentrated urine. Intravenous antibiotics was prescribed with oxygen therapy by facial mask. A conservative treatment of the fecaloma with manual disimpaction and bowel enemas was practiced but was unsuccessful. Surgical treatment was discussed with surgeon and anesthesiologist teams, and it was considered to have high operatory risk. Her family was informed but they refused the surgery. The patient's condition deteriorated, and she died on the third day of hospitalization from septic shock.

## Discussion

To the best of our knowledge, a huge fecaloma complicated by aspiration pneumonia has never been reported in the literature. Although it is an exceptional complication, physicians should consider it as a possible cause of pneumonia in older patients with chronic constipation. In our patient, treatment by manual disimpaction and bowel enemas was tried but it was inefficient. An endoscopic treatment could be an interesting alternative but was not available in our institution, so was a limitation in the therapeutic management in this case. The surgery was rejected because of high operatory risk. A fatal outcome may be the consequence of delayed extraction of the fecal impaction by life-threatening complications, as in our case.

Fecaloma is a hard stool impaction in the rectum and/or colon that cannot be evacuated spontaneously. ^3, 4^ It typically occurs in the rectum and sigmoid colon. Cecal and small bowel localizations are rarely reported. ^6, 7^ Fecaloma is typically seen in elderly patients, particularly in those bed bound and institutionalized and more common in women. ^3– 5^ Chronic constipation, frequently seen in elderly and bedridden patients, plays an important role in the development of fecaloma ^1, 3, 4, 8^ Other common risk factors reported in the literature were neuropsychiatric diseases and use of constipating medication like antiepileptic, antidepressant and opioid therapy. ^1, 2, 7, 9– 12^ Fecaloma was also associated with Chagas disease, Hirschsprung's disease, inflammatory and neoplastic diseases, scleroderma, diabetic neuropathy, and anorectal malformations. ^2, 4, 7^

The fecaloma’s symptoms are various and non-specific. The most common complaints are constipation, abdominal pain, abdominal distention, nausea and vomiting. ^3– 5^ Fecaloma can be also be revealed by rectal bleeding, urinary retention and respiratory insufficiency, febrile status, impaired consciousness, altered general condition ^7, 13, 14^ Physical examination can demonstrate an abdominal distension, tenderness or mass on palpation and a hard mass in the rectum in digital rectal examination. ^7, 10, 14^ In our case, respiratory signs including hypoxemia and pneumonia were the predominant features. The fecaloma was also responsible of urinary retention with renal failure. Fecaloma diagnosis is made radiologically through X-rays, barium enema, abdominal ultrasound, or abdominal CT scans. ^1, 2^

Complications associated with fecal impaction are estimated at 40.6% of cases. ^3^ Some of these complications can be life threatening. ^7, 14^ Colonic complications, secondary to the effect of the fecaloma on the intestinal wall or lumen, leads to several injuries like bowel obstruction, ^2, 15^ Intussusception, ^16^ stercoral ulcer, ^1, 11^ stercoral colitis ^8^ or toxic megacolon leading to colonic perforation and fecal peritonitis and bleeding ^2, 3, 7^ Otherwise, the fecal mass can compress adjacent anatomical structures and lead to hydronephrosis, ^12, 14, 17^ nerve compression and deep vein thrombosis. ^2^

The fecaloma’s management is sometimes challenging. Therapeutic options include conservative, endoscopic and surgical treatments. The conservative treatment of fecalomas, especially those located in the rectum, consists of manual disimpaction of the fecal mass associated with the administration of laxatives and enemas to soften the hardened stool. ^12– 14^ Oral lavage with polyethylene glycol solution in sometimes helpful in case of proximal fecal impaction. ^4^ Successful management of fecaloma by an endoscopic fragmentation of fecal material in the distal colon has been recently reported. ^9, 11^ When conservative treatment fails, surgical intervention is required to prevent complications. Surgery should be indicated first-line in case of giant fecaloma, and when the patient’s general condition is poor or peritonism signs are present. ^4, 10, 18^ Fecaloma formation must be prevented in high risk patients with laxatives, fibers and water intake. ^4^ Some authors propose routine abdominal radiological exploration for these patients to prevent the development and irreversible complications. ^1, 2^

When it is not treated promptly with the best treatment method, fecaloma can be life-threatening. ^7^ In fact, patients presenting with fecal impaction are considered to be at high risk for serious morbidities and mortality reported in 40.6% and 21.9% patients, respectively. ^3^ The prognosis is particularly worse in elderly patients and in those with heart or neuropsychiatric diseases and chronic renal failure. ^4, 5^

## Conclusion

Fecaloma commonly reported in elderly patients suffering from chronic constipation, should be considered as a serious condition. Early diagnosis should be made to relieve symptoms and prevent complications which can be often serious. The diagnosis should be suspected when there are signs of organ compression, especially in elderly patients with chronic constipation. Aspiration pneumonia can be one of the fecaloma’s complications.

## Data availability

All data underlying the results are available as part of the article and no additional source data are required.

## Consent

Written informed consent for publication of their clinical details and clinical images was obtained from the family of the patient.

## References

[1] Huang W-C Huang T-Y Chen P-J : Fecaloma impaction and stercoral ulcer. *Adv Dig Med.* 2020;7(3):166–169. 10.1002/aid2.13176

[2] Gil AG Liu Q Ho S : Giant Fecaloma Causing Large Bowel Obstruction: A Case Report. *Gastro Med Res.* 2020;5(1).GMR.000602. 10.31031/GMR.2020.05.000602

[3] Sommers T Petersen T Singh P : Significant morbidity and mortality associated with fecal impaction in patients who present to the emergency department. *Dig Dis Sci.* 2019;64(5):1320–1327. 10.1007/s10620-018-5394-8 30535766 PMC6499648

[4] Serrano Falcón B Barceló López M Mateos Muñoz B : Fecal impaction: a systematic review of its medical complications. *BMC Geriatr.* 2016;16:4. 10.1186/s12877-015-0162-5 26754969 PMC4709889

[5] Halawi HM Maasri KA Mourad FH : Faecal impaction: in-hospital complications and their predictors in a retrospective study on 130 patients. *Colorectal Dis Off J Assoc Coloproctology G B Irel. févr* .2012;14(2):231–236. 10.1111/j.1463-1318.2011.02769.x 21848667

[6] Wang BT Lee SY : Cecal fecaloma: A rare cause of right lower quadrant pain. *Eur J Radiol Open.* 2019;6:136–138. 10.1016/j.ejro.2019.03.006 30989091 PMC6449651

[7] Blakaj F Hamza A Bicaj B : Giant fecaloma mimicking large tumor of the abdomen: A case report. *Forensic Sci Int Rep.* 2020;2:100108. 10.1016/j.fsir.2020.100108

[8] Abasin T Din MN : Stercoral colitis due to massive fecal impaction: a case report and literature review. *Radiol Case Rep.* 2021;16:1946–1950. 10.1016/j.radcr.2021.04.067 34149980 PMC8193071

[9] Ghosh G Shah S Maltz C : A case of a giant fecaloma. *Clin Gastroenterol Hepatol.* 2018;16(4):e48. 10.1016/j.cgh.2017.08.004 28804033

[10] Khan MA Dar HA Shah AH : Fecaloma presenting as huge abdominal mass. *JGH Open.* 2020;4(2):294–295. 10.1002/jgh3.12221 32280783 PMC7144756

[11] Moraveji S Siddiqui AD : Unique Endoscopic Therapy of Colonic Obstruction Caused by a Fecaloma: 1631. *Off J Am Coll Gastroenterol ACG.* 2018;113(Supp 938).

[12] Tu K-C Kuo J-R : Fecaloma causing megacolon and bilateral hydronephrosis. *Formos J Surg.* 2020;53(2):70. 10.4103/fjs.fjs_58_19

[13] Curro G Lazzara C Latteri S : Supergiant fecaloma as manifestation of chronic constipation. *Il G Chir.* 2017;38(1):53. 10.11138/gchir/2017.38.1.053 28460206 PMC5730403

[14] Ozlu DN Seker KG Seker YC : Postrenal Acute Renal Failure Due to Giant Fecaloma-related Bilateral Hydronephrosis: A Case Report and Brief Literature Review. *Cureus.* 2020;12(4). 10.7759/cureus.7815 32467791 PMC7249776

[15] Kalayci T Genel IDH : A Rare Ileus Etiology: Giant Fecaloma. *Causapedia.* 2018;7(3):164–168.

[16] Khan Z Darr U Renno A : Transient descending colocolonic intussusception due to a large fecaloma in an adult. *ACG Case Rep J.* 2017;4. 10.14309/crj.2017.94 28798942 PMC5541757

[17] Joo N Lee HS : Acute Hydronephrosis owing to A Giant Fecaloma in an Older Patient. *Ann Geriatr Med Res.* 2020;24(3):223. 10.4235/agmr.20.0052 32933229 PMC7533188

[18] Tchangai B Alassani F Tchaou M : Staged Surgery for Giant Fecaloma Complicating Idiopathic Megacolon. *Open J Gastroenterol.* 2016;6(12):418–422. 10.4236/ojgas.2016.612044

