# Supplementary material for: Case Report: A giant fecaloma revealed by severe aspiration pneumonia and urinary retention
Source: F1000Res. 2021 Jul 29;10:691. [Version 1] doi: 10.12688/f1000research.54855.1 (PMC11069041; doi:10.12688/f1000research.54855.1)
Supplement: Peer review history

# Reviewer report: Reviewer response for version 1

**DOI:** 10.5256/f1000research.58376.r264316

**Published:** 2024-05-03

**Authors:** Noboru Misawa

**Affiliations:** 1 Yokohama City University, Yokohama, Japan

In this paper entitled “Case Report: A giant fecaloma revealed by severe aspiration pneumonia and urinary retention”, the authors has reported a case of a giant fecaloma revealed by severe aspiration pneumonia and urinary retention. I would like to add a comment.

 Major comment

1. In this report, do the authors think that aspiration pneumonia was caused by fecaloma? If so, the authors need to be more specific about the mechanism.
2. The authors stated that in the Discussion, “Fecaloma diagnosis is made radiologically through X-rays, barium enema, abdominal ultrasound, or abdominal CT scans”. However, more specific information should be given on diagnostic methods for the assessment of faecal masses in the recto-colon. In particular, the recent report on ultrasound assessment of faeces in the rectum should be mentioned.

Are enough details provided of any physical examination and diagnostic tests, treatment given and outcomes?

Partly

Is the case presented with sufficient detail to be useful for other practitioners?

Yes

Is sufficient discussion included of the importance of the findings and their relevance to future understanding of disease processes, diagnosis or treatment?

Yes

Is the background of the case’s history and progression described in sufficient detail?

Yes

Reviewer Expertise:

constipation

I confirm that I have read this submission and believe that I have an appropriate level of expertise to confirm that it is of an acceptable scientific standard.

# Reviewer report: Reviewer response for version 1

**DOI:** 10.5256/f1000research.58376.r101553

**Published:** 2021-12-21

**Authors:** Hyung Seok Lee

**Affiliations:** 1 Division of Nephrology, Department of Internal Medicine, Hallym University Sacred Heart Hospital, Anyang, South Korea

This case report deals with complications caused by chronic constipation that is frequently encountered but often overlooked in the elderly.

 Complications caused by giant fecaloma can occur in various ways by compressing or displacing adjacent organs as well as direct harm, such as bowel wall perforation. In particular, this case report presents that if fecaloma is not timely treated, it can lead to a rare complication, such as aspiration pneumonia, which has useful clinical implications for other practitioners.

 This article is well written and contains enough information. Therefore, I have no further comments on this article.

Are enough details provided of any physical examination and diagnostic tests, treatment given and outcomes?

Yes

Is the case presented with sufficient detail to be useful for other practitioners?

Yes

Is sufficient discussion included of the importance of the findings and their relevance to future understanding of disease processes, diagnosis or treatment?

Yes

Is the background of the case’s history and progression described in sufficient detail?

Yes

Reviewer Expertise:

Interventional Nephrology

I confirm that I have read this submission and believe that I have an appropriate level of expertise to confirm that it is of an acceptable scientific standard.
